# Associations Between Cognitive Performance and Motor Signs in Older Adults with Alzheimer’s Dementia

**DOI:** 10.3390/medicina61122116

**Published:** 2025-11-27

**Authors:** Ioannis Liampas, Vasileios Siokas, Chrysoula Marogianni, Antonia Tsika, Metaxia Dastamani, Polyxeni Stamati, Efthimios Dardiotis

**Affiliations:** Department of Neurology, University Hospital of Larissa, School of Medicine, University of Thessaly, 41100 Larissa, Greece; vsiokas@med.uth.gr (V.S.); c.marogianni@gmail.com (C.M.); antonellatsi@hotmail.com (A.T.); mdastamani@yahoo.gr (M.D.); tzeni_0@yahoo.gr (P.S.); edar@med.uth.gr (E.D.)

**Keywords:** processing speed, executive function, bradykinesia, gait disorder, postural instability

## Abstract

*Background and Objectives:* The interplay between motor tasks and cognition in Alzheimer’s dementia (AD) remains insufficiently characterised. We hypothesised that prefrontal-mediated cognitive functions could contribute to motor impairments in older adults with AD. *Materials and Methods:* Cross-sectional data from the National Alzheimer’s Coordinating Centre (NACC) were analysed. Our sample included older adults (≥60 years) with a baseline diagnosis of AD. The Unified Parkinson’s Disease Rating Scale Part-III was used to assess the presence or absence of motor signs. Episodic memory, language, confrontation naming, attention, processing speed, and executive function were assessed using a neuropsychological battery. Binary logistic models examined the relationship between cognitive performance and motor manifestations. *Results*: Of 44,713 NACC participants, 5124 individuals with complete covariate data were included in the analysis, 1339 with and 3785 without motor signs. Participants were predominantly female (~55%), with an average age of 76.5 ± 7.9 years and mean education of 14.2 ± 3.7 years. The presence of motor manifestations was related to slower processing speed (Trail Making Test—Part A) and impaired executive function (Trail Making Test—Part B). No covariate modified these associations. Among specific motor domains, impaired chair rise was related to executive dysfunction, whereas postural instability, impaired posture–gait, and bradykinesia were related to slower mental processing. Hypophonia, masked facies, resting tremor, action–postural tremor and rigidity were not associated with any cognitive measure. *Conclusions*: Processing speed and, to a lesser extent, executive function emerged as the main cognitive functions associated with motor manifestations in older adults with AD. Further research is needed to clarify the nature of this association, including potential causal pathways.

## 1. Introduction

In alignment with cognitive decline [1], motor deterioration becomes an increasingly burdensome issue with ageing [2]. Numerous studies have investigated the neural correlates that mediate the relationship between motor and cognitive decline across ageing. Synaptic changes, cerebrovascular insults, and neurodegenerative alterations appear to affect both functions [3,4]. Of note, these neural correlates may contribute to motor impairment either directly [5] or indirectly, i.e., through cognitive impairment, reflecting that motor performance is heavily based on high-level mental processing [6].

In extrapyramidal movement disorders, impairments in prefrontal-mediated cognitive functions, such as executive dysfunction and slower mental processing, have been found to contribute significantly to Parkinsonian symptoms [7,8,9,10,11]. Far less is known about the interdependence between motor tasks and cognition in Alzheimer’s dementia (AD). Theoretically, similar cognitive deficits could underlie Parkinsonian features and gait–balance disorders in AD. Nevertheless, unalike PD, the neuropsychological profile of AD is primarily characterised by memory impairment, often followed by the progressive emergence of non-memory cognitive symptoms [11,12,13,14]. Prefrontal-mediated cognitive dysfunction occurs as well, typically later in the disease course [15]. Of note, this temporal progression parallels the incidence of motor manifestations, which tend to arise during the later stages of AD [16,17,18].

Based on the evidence outlined above, we hypothesised that prefrontal-mediated cognitive impairments may also contribute to motor dysfunction in AD. The prefrontal cortex plays a central role in both executive control and motor planning, integrating attention, mental processing, working memory, and goal-directed action [19]. Degeneration or disconnection within these frontal networks—especially within fronto-striatal and fronto-parietal pathways—may contribute simultaneously to slower processing speed, executive dysfunction, and impaired motor performance [20,21]. This cognitive–motor coupling framework posits that prefrontal-mediated deficits disrupt the top-down modulation of motor output, leading to observable signs such as bradykinesia, postural instability, and gait disturbance with higher-order cognitive decline in dementia [18].

Previous research has scarcely explored this theory in AD. Moreover, most studies focused on gait–balance disorders and falls, while authors have failed to account for several crucial confounders in the analytic plan [18]. Therefore, the aim in undertaking the current study was to explore the relationship between cognitive performance and motor manifestations in older adults with AD. We hypothesised that prefrontal-mediated cognitive impairments (i.e., slower processing speed and/or executive dysfunction) would be related to motor impairments in AD. We leveraged data from the Uniform Data Set (UDS) and conducted a cross-sectional analysis involving older adults with AD. Our findings could potentially have implications for phenotypic subgroup definition and precision medicine.

## 2. Materials and Methods

The current analysis was based on baseline data from the UDS. The UDS is a central repository of prospectively collected data from multiple National Institute on Aging/National Institutes of Health (NIA/NIH)-funded Alzheimer’s Disease Centres (ADCs) across the United States [22]. It is stewarded by the National Alzheimer’s Coordinating Centre (NACC) and is freely available to research scientists upon request (https://naccdata.org/). The rationale and the key methodological features of the UDS have been detailed elsewhere [23,24,25]. In brief, study procedures were approved by the Institutional Review Boards overseeing each ADC and participants granted informed consent prior to participation. Enrolment–recruitment processes may differ among participating ADCs: participating volunteers may actively pursue professional consultation, may be referred by other clinicians or family members, may be actively recruited, etc. After recruitment, they are evaluated by trained personnel using uniform, standardised, multidisciplinary assessments, on an approximately yearly basis. Among others, the UDS assembles demographic and anthropometric data, neuropsychological, neuropsychiatric, clinical and functional evaluations, family and personal medical history, and available laboratory investigations. Depending on the specific protocol of each ADC, neurocognitive diagnoses were established by either an interdisciplinary consensus team (in most cases) or a single clinician (who examined the participant), using standard clinical criteria [26,27,28,29]. Biomarkers were not available in first two versions of the UDS.

The present study was based on baseline, cross-sectional NACC data from a total of 46 ADCs, from the inception of the UDS (September 2005) to the December 2022 data freeze. We focused on baseline evaluations of adults older than 60 years of age with a diagnosis of AD. Individuals with a concomitant diagnosis of PD or a Parkinsonian syndrome were excluded. Participants being treated with anti-Parkinsonian medications, irrespective of the underlying diagnosis, were also excluded.

### 2.1. Measurement of Motor Signs

Motor signs were assessed on the Unified Parkinson’s Disease Rating Scale Part-III (UPDRS-III). UPDRS-III was administrated in the first two versions of the UDS, up to 2015. It consists of 27 items. These items were clustered into 9 domains of motor function, as follows [30]: (1) hypophonia (single item); (2) masked facies (single item); (3) resting tremor (combined five items regarding tremor at rest in the face/lips/chin and four extremities); (4) action/postural tremor (combined two items regarding tremor at rest in the hands); (5) rigidity (combined five items regarding rigidity in the neck and four extremities); (6) bradykinesia (combined nine items: bilateral finger tapping, hand movements, rapid alternating movements of the hands, leg agility, and body bradykinesia); (7) impaired chair rise (single item); (8) impaired posture–gait (combined two items: posture and gait); and (9) postural instability (single item). The clustering of UPDRS-III items into motor domains was guided by factor-analytic findings from previous studies and clinical considerations, ensuring that the resulting clusters were both empirically supported and clinically meaningful [31,32].

Each item was treated as a dichotomous variable: absent (score < 2) or present (score ≥ 2). The rationale for this threshold has been detailed before [33,34]. In turn, nine dichotomous motor variables (motor domains) were devised, such that participants were said to have a motor sign if they scored ≥ 2 in at least one of the items of the respective motor domain [35]. A global dichotomous motor variable was also created, according to which participants were divided into those with (if they scored ≥ 2 in at least one motor domain) and those without (if they scored < 2 in all motor domains) motor manifestations.

### 2.2. Measurement of Cognitive Performance

The first two versions of the UDS focus on the following cognitive domains: episodic memory, language, confrontation naming, attention, processing speed, and executive function. Episodic memory (immediate and delayed recall) was assessed on the Logical Memory Test (Story A) from the Wechsler Memory Scale—Revised (WMS-R) [36], language on the total word production summing the animal and vegetable fluency tasks [37], confrontation naming according to the 30-item version of the Boston Naming Test (BNT-30) [38], attention using the Digit Span Test (DST, forward and backward conditions) from the WMS-R [36], processing speed on the Trail Making Test—Part A (TMT-A), and executive function on the Trail Making Test—Part B (TMT-B) [39]. The administration and scoring of these tests have been previously detailed [40]. Higher scores reflect better cognition in the majority cases, except for the following: higher scores in TMT-A and TMT-B reflect worse performance.

### 2.3. Covariates Considered

Participant age at visit (years), education (years of formal schooling), and Geriatric Depression Scale (GDS) scores were treated as scale variables. Sex (male/female), race (Caucasian, African American, Asian, other), *APOE* genotype [APOE3 group (*APOE3/3*)*,* APOE2 group (*APOE2* carriers)*,* APOE4 group (*APOE3/4*, *APOE4/4*)], global Clinical Dementia Rating (CDR) scores (0.5, 1.0, 2.0, 3.0—reflecting the stage of AD), neuropsychiatric severity scores (NPSs—no, mild, moderate/severe), and medication intake (four separate dichotomous variables, see below) were treated as categorical variables. Medication intake focused on antidepressants [including selective serotonin reuptake inhibitor (SSRIs), serotonin and norepinephrine reuptake inhibitors (SNRIs), tricyclic, tetracyclic, monoamine oxidase inhibitors (MAOIs), and miscellaneous (e.g., bupropion, trazodone, mirtazapine, 5-hydroxytryptophan,)], anxiolytics [including benzodiazepines, barbiturates, and other sedatives and hypnotics (e.g., buspirone, hydroxyzine, zolpidem, melatonin)], antipsychotic agents [including phenothiazines, thioxanthenes, atypical antipsychotics, combinations with antipsychotics (e.g., amitriptyline–chlordiazepoxide or perphenazine), and other miscellaneous agents (e.g., lithium)], and FDA-approved medication for AD (tacrine, donepezil, rivastigmine, galantamine, and memantine) [16].

NPS was evaluated using data from the Neuropsychiatric Inventory Questionnaire (NPI-Q) (Cummings et al., 1994) [41]. NPI-Q evaluates 12 domains (delusions, hallucinations, agitation/aggression, depression/dysphoria, anxiety, elation/euphoria, apathy/indifference, disinhibition, irritability/lability, aberrant motor behaviour, night-time behaviours, and appetite/eating) according to a 4-point severity scale: no; mild (noticeable, but not a significant change); moderate (significant, but not a dramatic change); or severe (very marked or prominent; a dramatic change). For each NPI-Q domain, participants were grouped into three categories: 0—absent; 1—mild; 2—moderate and severe symptomatology (due to the small prevalence of moderate and severe symptoms). Sequentially, the global NPS was calculated as follows: 0—no symptoms; 1—at least one mild symptom with no moderate and/or severe symptoms; 2—at least one moderate and/or severe symptom.

### 2.4. Statistical Analysis

Eligible participants without any missing data per motor domain ranged between 3266 and 3334 (depending on the number of cases with missing data on each motor domain) (original datasets). To address potential attrition bias, we performed multiple data imputation in the subset of individuals without missing data on covariates (5124 participants without missing data on covariates but with missing data on neuropsychological performance and/or motor assessments). Missing data were imputed using SPSS’s automatic multiple imputation procedure. Prior to imputation, missingness patterns were examined and assumed to be missing at random (MAR), consistent with the default SPSS settings. The automatic multiple imputation algorithm in SPSS generated 5 datasets by replacing missing values with plausible estimates based on observed data, using fully conditional specification (FCS). This approach accounts for uncertainty due to missingness and preserves the variability and distribution of the original data. Imputed datasets were then pooled for subsequent analyses, following standard Rubin’s rules.

Adjusted logistic regression models were tested by motor manifestation: one using the original and one using the imputed datasets. Each model featured the aforementioned neuropsychological measurements and set of covariates. To address multicollinearity, we ensured that all predictors met conventional criteria, with variance inflation factors below 2.5 and tolerance values above 0.4, indicating low correlations among predictors. In order for an association to be counted as significant, results from both the original and imputed datasets had to be statistically significant.

An exploratory analysis was also conducted to assess potential interactions between covariates and cognitive functions. Specifically, we aimed to examine whether covariates modified the relationship between cognitive performance and the global motor variable (defined as the presence of at least one motor sign; dependent variable). Again, two adjusted logistic regression models were tested per covariate: one using the original and one using the imputed datasets. Each model featured the aforementioned set of covariates, the cognitive functions that exhibited significant associations in the main analysis (e.g., processing speed and executive function), and interaction terms (e.g., in the case of sex, sex by processing speed and sex by executive function interactions).

To account for potential misclassification of AD cases, we conducted a sensitivity analysis for the global motor variable, excluding participants with more severe dementia (CDR > 1). This approach was intended to enhance the clinical specificity for AD-related impairment by focusing on participants across early symptomatic stages where the clinical diagnostic criteria are more reliable. Again, two adjusted logistic regression models were tested: one using the original and one using the imputed datasets.

An additional sensitivity analysis was performed to ensure that the dichotomization of UPDRS-III scores did not distort associations. For this purpose, an adjusted univariate general linear model was tested, using the total UPDRS-III score (sum of 27 individual items) as a continuous dependent scale variable. Only the original dataset was analysed, as the multiple imputation procedure had been performed using dichotomised UPDRS-III scores and UPDRS-III values were not imputed as continuous (scale) variables.

All analyses were conducted using the IBM SPSS Statistics Software Version 27 (Chicago, IL, USA). Statistical significance was determined using a conventional α threshold of 0.05. To be considered statistically significant, an association was required to reach significance in both the original and imputed datasets. Effect sizes (odds ratios, ORs) and precision estimates (95% confidence intervals, 95%CIs) are provided. ORs reflect the change in odds of the outcomes (motor manifestation) per one-unit increase in the predictor variable (performance on cognitive task). Baseline differences between those with and without motor manifestations were analysed using (1) independent samples t-test (scale variables) and (2) Pearson’s chi-squared tests (categorical variables).

## 3. Results

**Figure 1:**
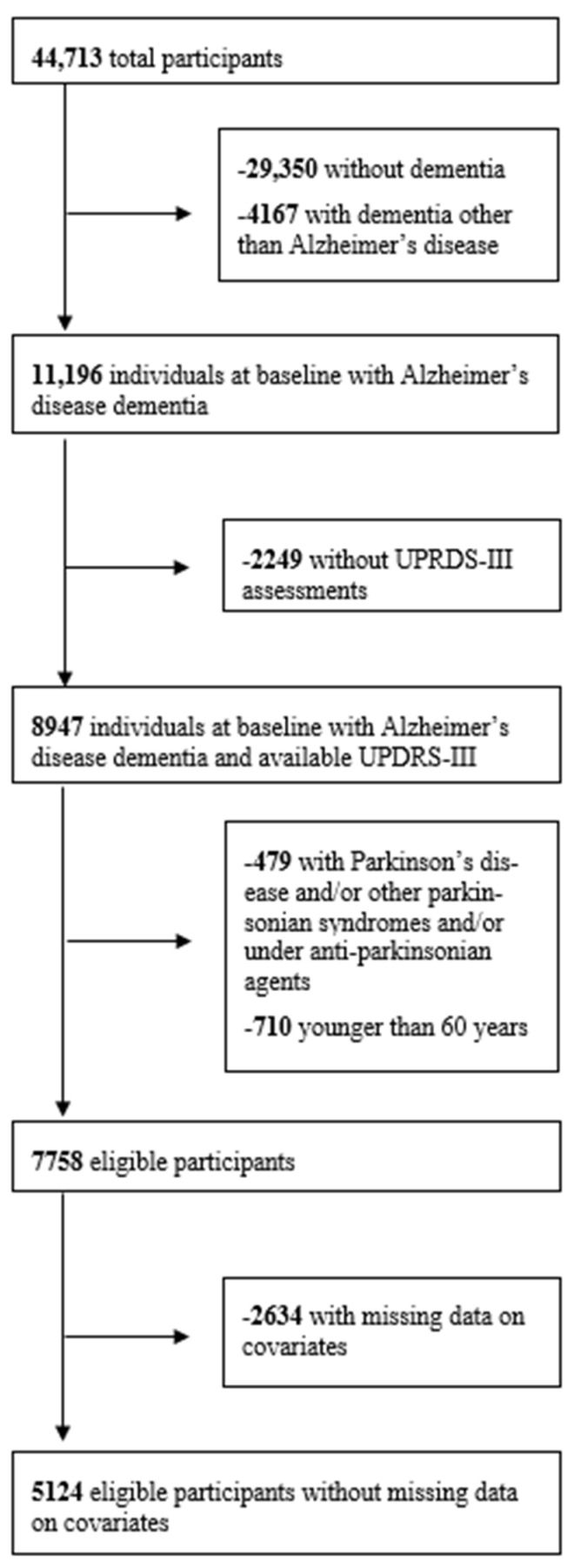
Participant flowchart.

There were 44,713 participants with at least one UDS evaluation. Of the 7758 eligible individuals, 5124 had no missing data on covariates (imputed subset). Among them, there were 1773 who had missing data on cognitive measurements. Depending on the number of cases with additional missing data on each motor domain (ranging from 17 to 85), the original subsets contained between 3266 and 3334 participants. The participant flowchart is in Figure 1.

Table 1 shows the characteristics of our sample. Participants with motor manifestations were older and less educated. Sex and race distributions were similar. *APOE4* carriage was more common in those without motor signs, whereas *APOE2* carriage was more common in those with motor signs. AD staging, depression scores, and neuropsychiatric burden were greater in the latter group. The use of psychotropic agents was more prevalent in the latter group, as well. As for cognitive scores, apart from delayed recall, those with motor manifestations performed worse on the remaining cognitive assessments (Table 2).

### Associations Between Cognitive Performance and Motor Signs in Older Adults with AD

The presence of motor manifestations (global motor variable) was related to processing speed and executive function (Table 3). We confirmed this finding using both the original and the imputed datasets. Associations were more prominent for processing speed (OR1 = 1.007 and OR2 = 1.005, based on the original and imputed datasets, correspondingly) than executive function (OR1 = 1.002 and OR2 = 1.002). For context, the aforementioned associations translate as follows: an OR of 1.007 and/or 1.002 would translate to 0.7% and/or 0.2% increased odds of motor symptoms per additional second required to complete the respective cognitive task, TMT-A for processing speed and/or TMT-B for executive function. Effects over multiple seconds are multiplicative (not additive). Therefore, when considering performance differences expressed in standard deviations, a 1-SD decrease in TMT-A (approximately 42 s), was associated with an approximate increase of 34% (1.007^42^) in the odds of motor manifestations; a 1-SD decrease in TMT-B (about 80 s) was associated with a 17% increase in the odds (1.002^80^).

**Figure 2:**
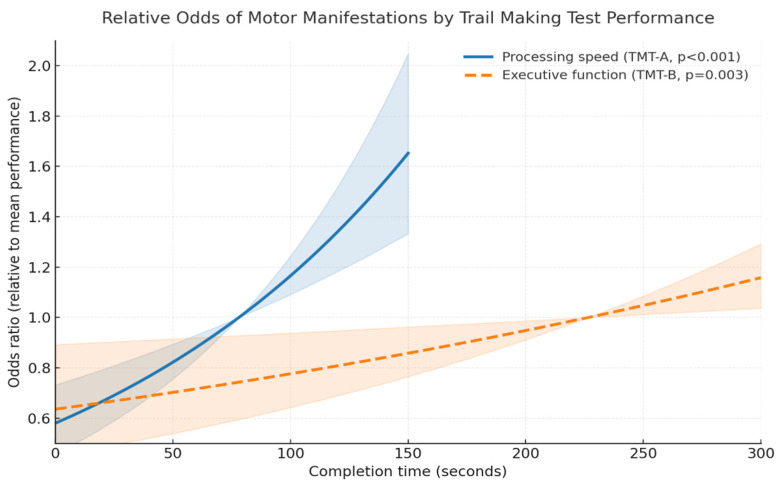
Predicted odds of having at least one motor sign as functions of performance on the TMT-A and TMT-B. The shaded regions indicate 95% confidence intervals.

To facilitate interpretation, Figure 2 shows the predicted odds ratios of having at least one motor sign as functions of performance on the TMT-A and TMT-B. Curves represent the change in odds of having at least one motor sign relative to the mean performance for each test, derived from adjusted logistic regression models. Longer completion times correspond to slower cognitive performance and are associated with progressively higher odds of motor signs.

The sensitivity analyses yielded almost identical results (Supplementary Tables S1 and S2): slower mental processing and executive dysfunction were related to motor manifestations in both analyses. The only difference from the main analysis was that both sensitivity analyses revealed an additional association between better episodic memory—delayed recall performance and motor signs. This finding failed to achieve statistical significance in the main analysis and might be trivial; however, it could also reflect that atypical cognitive phenotypes of AD, characterised by relatively preserved episodic memory, may be associated with motor signs.

Detailed results for individual motor domains are in Table 4. In brief, impaired chair rise was solely related to executive dysfunction (OR1 = 1.005 and OR2 = 1.004). Postural instability (OR1 = 1.006 and OR2 = 1.006), impaired posture–gait (OR1 = 1.007 and OR2 = 1.008), and bradykinesia (OR1 = 1.006 and OR2 = 1.007) were only related to slower processing speed (ORs 1 and 2 correspond to the original and imputed datasets). Hypophonia, masked facies, resting tremor, action–postural tremor, and rigidity were not associated with any cognitive assessment.

Finally, exploratory analyses were performed to investigate the potential modifying impact of covariates on the relationship of executive function and processing speed with the global motor variable (Table 5). No significant interactions emerged.

## 4. Discussion

In older adults with AD, the presence of motor manifestations was related to poorer performance on processing speed and executive function tasks. Among individual motor domains, impaired chair rise was related exclusively to executive dysfunction, whereas postural instability, impaired posture–gait, and bradykinesia were linked only to slower processing speed. Hypophonia, masked facies, resting tremor, action–postural tremor, and rigidity were not associated with any cognitive measure. None of the examined covariates modified these relationships.

Previous research in cognitively unimpaired adults has yielded similar results, demonstrating prominent associations between impaired chair rise and bradykinesia with executive function and processing speed [30]. Although evidence in AD populations is limited, one study reported an association between bradykinesia and frontal cognitive deficits, while another identified a contribution of executive dysfunction to postural instability and falls [42]. However, the paucity of relevant data prevents firm conclusions and further studies are needed to replicate and extend our findings.

Recognition of motor signs in AD carries important diagnostic, prognostic, and therapeutic implications. First, Parkinsonian signs are cardinal features of other dementia entities, and clinicians often rely on their presence to dismiss the diagnosis of AD [43]. However, a substantial proportion of patients with AD, without concomitant Lewy body pathology, may exhibit parkinsonian symptoms and signs [44]. The literature suggests that the proportion of individuals with Parkinsonian features increases over time, exhibiting a positive correlation with clinical progression and propagation of neuropathology [16,17,45]. In this context, our findings could have diagnostic implications and prompt additional diagnostic investigations in certain phenotypic subgroups, especially in patients diagnosed over the advanced clinical stages.

From a management standpoint, clinicians typically focus on the predominant non-motor aspects of AD [46]. Cognitive decline and neuropsychiatric symptoms are major contributors to caregiver burden and are often prioritised in both clinical care and clinical trials [47,48]. Our findings suggest that motor signs merit closer attention, particularly in patients with specific cognitive profiles. Identifying motor manifestations in such subgroups could improve individualised patient care. Moreover, the inclusion of motor outcomes in clinical trials—especially those investigating the dysexecutive variant of AD—could improve the characterization of disease progression and treatment response.

Although the exact pathophysiological underpinnings of motor signs in AD are not well-established, animal models have provided evidence of extracerebral β-amyloid accumulation in the spinal cord and skeletal muscles, which may have a direct deleterious effect on these tissues [49]. Additional mechanisms may involve neuronal loss in the primary and supplementary motor cortices, cerebellum, and basal ganglia, as well as synaptic dysfunction and corticospinal tract degeneration. Many patients are also likely exhibit mixed neuropathologies, including vascular lesions, white matter damage, α-synucleinopathy, or TDP-43 pathology, which may contribute to motor symptoms independently of AD pathology. In addition, certain motor signs, such as impaired chair rise, may also reflect general physical deconditioning commonly observed in older adults with AD.

This analysis has several weaknesses, including being cross-sectional and, thus, inappropriate to reveal causality. Future longitudinal studies ought to re-assess our findings and capture the true direction of these associations. Second, although several important covariates were considered, our findings may have been driven by residual confounding. Third, the NACC database may be subject to inherent selection bias, as participants tend to be volunteers with higher levels of education. Furthermore, in the majority of cases, cognitive diagnoses were based on clinical criteria; biomarkers were not uniformly available. Therefore, there may have been a misclassification of other neurodegenerative conditions, suc as AD. Future research ought to enrol individuals with biomarker-supported clinical diagnoses so that the diagnostic accuracy is improved. Biomarker investigations may further enhance our understanding of the interdependence between neuropathology, cognitive dysfunction, and motor signs in older adults with AD. Moreover, the count of some motor signs (hypophonia, resting tremor, masked facies) was very small, leading to a relative lack of statistical power in the respective analyses. Additionally, considering the number of analyses performed, there is a risk of false positive findings. Of note, this risk does not apply to the main analysis (the conventional cut-off α = 0.05 should be considered for the global motor variable). As for the subdomain-specific analyses, the larger-imputed datasets generated highly significant results (*p* < 0.001 in three analyses and *p* = 0.011 in one), indicating very strong statistical evidence against the null hypothesis. Therefore, we do believe that the associations captured are non-trivial. Finally, processing speed and executive function are correlated and may represent overlapping cognitive constructs. This conceptual overlap is well-recognised in the literature, as both domains rely on shared neural and cognitive mechanisms. However, we assessed potential multicollinearity and found that the correlation between these variables did not reach a level that would compromise model stability. Therefore, both domains were retained in the analyses to preserve theoretical completeness and comparability with prior studies.

## 5. Conclusions

This study demonstrates that, in older adults with AD, slower processing speed and executive dysfunction are significantly associated with the presence of motor manifestations. Impaired chair rise was strongly associated with executive dysfunction, whereas postural instability, impaired posture–gait, and bradykinesia were strongly linked to slower processing speed. These findings should not be interpreted as causational due to the cross-sectional design. Longitudinal and biomarker-supported studies are warranted to elucidate underlying neural mechanisms.

## Figures and Tables

**Table 1:** Participants’ characteristics.

Variable	*No Motor Sign* (*N* = 3785)	*At Least One Motor Sign* (*N* = 1339)	*p*-Value
Age (years)	75.4 ± 7.7	79.5 ± 7.9	*p* < 0.001
Sex (male/%)	1716/45.3%	588/43.9%	*p* = 0.368
Education (years)	14.3 ± 3.6	13.8 ± 3.9	*p* < 0.001
Race (Caucasian/African American/Asian/other)	3198/446/50/91 (84.5/11.8/1.3/2.4%)	1103/164/29/43 (82.4/12.2/2.2/3.2%)	*p* = 0.051
*APOE* genotype (e3e3/e3e4/e3e2/e4e4/e4e2/e2e2)	1316/1658/148/544/113/6 (34.8/43.7/3.9/14.4/3.0/0.2%)	552/522/88/135/39/3 (41.2/39.0/6.6/10.1/2.9/0.2%)	*p* < 0.001
GDS (15)	2.4 ± 2.5	2.8 ± 2.8	*p* < 0.001
NPS (none/mild/moderate or severe)	646/1323/1816 (17.0/35.0/48.0%)	184/427/728 (13.7/31.9/54.4%)	*p* = 0.001
Global CDR (0.5/1.0/2.0/3.0)	1471/1852/423/39 (38.9/48.9/11.2/1.0%)	294/631/337/77 (22.0/47.1/25.2/5.7%)	*p* < 0.001
FDA approved drugs for AD	2685/70.9%	935/69.8%	*p* = 0.443
Anxiolytics (Yes/%)	299/7.9%	137/10.2%	*p* = 0.009
Antidepressants (Yes/%)	1308/34.6%	525/39.2%	*p* = 0.002
Antipsychotics (Yes/%)	139/3.7%	111/8.3%	*p* < 0.001

Scale variables are presented in mean ± standard deviation; categorical variables are presented in absolute numbers (%); *N*: number of individuals; *APOE*: *apolipoprotein*; GDS: geriatric depression scale; NPS: neuropsychiatric score; CDR: clinical dementia rating scale; AD: Alzheimer’s dementia.

**Table 2:** Participants’ cognitive performance. Measurement units for processing speed (TMT-A) and executive function (TMT-B) are “seconds”.

Variable	*No Motor Sign*	*At Least One Motor Sign*	*p*-Value
Original dataset			
Episodic memory—immediate recall	4.2 ± 3.5	3.7 ± 3.4	*p* < 0.001
Episodic memory—delayed recall	1.9 ± 2.9	1.8 ± 2.9	*p* = 0.251
Attention	9.6 ± 2.1	9.1 ± 2.2	*p* < 0.001
Processing speed	67 ± 40	84 ± 42	*p* < 0.001
Executive function	207 ± 87	238 ± 79	*p* < 0.001
Semantic verbal fluency	11.0 ± 5.0	9.3 ± 5.2	*p* < 0.001
Confrontation naming	19.7 ± 7.1	17.9 ± 7.2	*p* < 0.001
Imputed datasets			
Episodic memory—immediate recall	4.2 ± 3.5	3.8 ± 3.4	*p* < 0.001
Episodic memory—delayed recall	1.9 ± 3.0	1.9 ± 2.9	*p* = 0.561
Attention	9.6 ± 2.2	9.0 ± 2.7	*p* < 0.001
Processing speed	69 ± 40	87 ± 42	*p* < 0.001
Executive function	214 ± 90	239 ± 80	*p* < 0.001
Semantic verbal fluency	11.0 ± 5.0	9.2 ± 5.3	*p* < 0.001
Confrontation naming	19.6 ± 7.2	17.5 ± 7.4	*p* < 0.001

Cognitive scores are presented in mean ± standard deviation.

**Table 3:** Associations between cognitive performance and the presence of motor signs in older adults with Alzheimer’s dementia. Measurement units for processing speed (TMT-A) and executive function (TMT-B) are “seconds”.

Original Dataset			
Cognitive Domain	Odds Ratio	95% Confidence Interval	*p*-Value
Episodic memory—immediate recall	1.003	0.964–1.043	*p* = 0.900
Episodic memory—delayed recall	1.042	0.999–1.085	*p* = 0.054
Attention	0.993	0.942–1.046	*p* = 0.788
**Processing speed**	**1.007**	**1.004**–**1.010**	***p* < 0.001**
**Executive function**	**1.002**	**1.001**–**1.004**	***p* = 0.003**
Semantic verbal fluency	1.002	0.978–1.027	*p* = 0.877
Confrontation naming	1.013	0.996–1.031	*p* = 0.138
**Imputed datasets**			
**Cognitive domain**	**Odds Ratio**	**95% Confidence Interval**	***p*** **-Value**
Episodic memory—immediate recall	1.007	0.976–1.040	*p* = 0.664
Episodic memory—delayed recall	1.049	1.012–1.086	*p* = 0.009
Attention	0.961	0.924–1.000	*p* = 0.051
**Processing speed**	**1.006**	**1.003**–**1.008**	***p* < 0.001**
**Executive function**	**1.002**	**1.000**–**1.003**	***p* = 0.034**
Semantic verbal fluency	0.991	0.972–1.011	*p* = 0.381
Confrontation naming	1.018	1.004–1.031	*p* = 0.009

Adjusted for participants’ age, sex, education, race, *APOE* genotype, depression scores, CDR scores, NPS scores, and medication intake. **Bold** denotes statistical significance.

**Table 4:** Associations of cognitive performance with motor manifestations. Odds ratios (ORs) with precision estimates [95% confidence interval lower (LL) and upper limits (UL)] are presented. Measurement units for processing speed (TMT-A) and executive function (TMT-B) are “seconds”.

Cognitive Domain	Original Dataset				Imputed Datasets			
	OR	LL	UL	*p* Value	OR	LL	UL	*p* Value
Impaired chair rise								
Immediate recall	1.029	0.961	1.103	0.412	1.036	0.981	1.094	0.206
Delayed recall	1.052	0.977	1.132	0.177	1.035	0.974	1.099	0.268
Attention	1.069	0.978	1.169	0.140	0.983	0.921	1.048	0.591
Processing speed	1.005	1.000	1.010	0.053	1.004	1.001	1.008	0.017
Executive function	**1.005**	**1.002**	**1.008**	**<0.001**	**1.004**	**1.002**	**1.006**	**0.001**
Verbal fluency	0.986	0.945	1.030	0.536	0.996	0.963	1.03	0.812
Confrontation naming	1.017	0.987	1.048	0.274	1.024	1.001	1.047	0.042
Impaired posture-gait								
Immediate recall	1.058	0.982	1.140	0.136	1.059	0.995	1.127	0.070
Delayed recall	0.992	0.914	1.077	0.848	0.992	0.923	1.066	0.826
Attention	1.047	0.953	1.150	0.343	0.981	0.917	1.049	0.566
Processing speed	**1.007**	**1.001**	**1.012**	**0.014**	**1.008**	**1.003**	**1.012**	**0.001**
Executive function	1.001	0.998	1.004	0.431	1.000	0.998	1.002	0.871
Verbal fluency	1.010	0.965	1.058	0.660	1.005	0.974	1.038	0.753
Confrontation naming	0.986	0.955	1.018	0.386	1.007	0.982	1.032	0.597
Postural instability								
Immediate recall	1.022	0.948	1.102	0.573	0.996	0.933	1.064	0.910
Delayed recall	1.025	0.946	1.111	0.547	1.046	0.977	1.120	0.195
Attention	0.961	0.873	1.057	0.414	0.93	0.863	1.003	0.059
Processing speed	**1.006**	**1.001**	**1.012**	**0.023**	**1.006**	**1.001**	**1.010**	**0.011**
Executive function	1.001	0.998	1.004	0.389	1.000	0.998	1.003	0.718
Verbal fluency	0.973	0.927	1.020	0.251	0.975	0.941	1.011	0.169
Confrontation naming	1.025	0.992	1.058	0.140	1.038	1.009	1.068	0.012
Bradykinesia								
Immediate recall	1.050	0.988	1.115	0.115	1.036	0.989	1.085	0.140
Delayed recall	1.031	0.968	1.098	0.339	1.047	0.993	1.105	0.088
Attention	0.940	0.868	1.017	0.125	0.943	0.892	0.997	0.040
Processing speed	**1.006**	**1.001**	**1.010**	**0.010**	**1.007**	**1.004**	**1.010**	**<0.001**
Executive function	1.002	1.000	1.004	0.083	1.001	0.999	1.003	0.169
Verbal fluency	1.008	0.971	1.046	0.684	0.985	0.955	1.017	0.361
Confrontation naming	0.992	0.966	1.019	0.576	1.003	0.985	1.021	0.763
Rigidity								
Immediate recall	1.051	0.972	1.136	0.212	1.047	0.987	1.112	0.130
Delayed recall	1.011	0.932	1.098	0.786	1.013	0.945	1.085	0.716
Attention	1.032	0.930	1.145	0.555	0.947	0.879	1.021	0.155
Processing speed	1.006	1.000	1.012	0.042	1.004	0.998	1.009	0.164
Executive function	1.002	0.999	1.005	0.156	1.001	0.998	1.004	0.474
Verbal fluency	0.970	0.923	1.019	0.220	0.968	0.933	1.005	0.087
Confrontation naming	1.020	0.985	1.056	0.260	1.017	0.992	1.042	0.182
Action-postural tremor								
Immediate recall	0.960	0.888	1.037	0.295	0.955	0.896	1.019	0.166
Delayed recall	1.066	0.986	1.151	0.107	1.058	0.989	1.131	0.104
Attention	0.970	0.874	1.077	0.570	0.952	0.881	1.028	0.208
Processing speed	0.999	0.992	1.005	0.708	1.001	0.996	1.006	0.799
Executive function	1.002	1.000	1.005	0.102	1.002	0.999	1.004	0.200
Verbal fluency	1.001	0.956	1.048	0.968	1.006	0.968	1.046	0.752
Confrontation naming	1.035	0.998	1.072	0.061	1.041	1.013	1.071	0.005
Resting tremor								
Immediate recall	1.055	0.930	1.196	0.407	1.056	0.933	1.195	0.380
Delayed recall	1.025	0.897	1.172	0.714	1.030	0.907	1.168	0.647
Attention	1.009	0.857	1.187	0.918	1.046	0.928	1.177	0.463
Processing speed	1.007	0.998	1.016	0.134	1.003	0.995	1.010	0.492
Executive function	0.999	0.994	1.004	0.647	1.001	0.996	1.005	0.706
Verbal fluency	0.959	0.885	1.040	0.311	0.970	0.905	1.039	0.380
Confrontation naming	0.989	0.935	1.045	0.695	1.001	0.959	1.046	0.950
Masked facies								
Immediate recall	1.020	0.896	1.162	0.763	1.091	0.989	1.202	0.081
Delayed recall	1.064	0.936	1.210	0.343	1.041	0.940	1.152	0.442
Attention	1.066	0.899	1.264	0.461	0.975	0.833	1.141	0.739
Processing speed	1.007	0.998	1.017	0.125	1.005	0.997	1.012	0.212
Executive function	1.002	0.997	1.007	0.337	1.002	0.996	1.008	0.437
Verbal fluency	1.008	0.932	1.091	0.834	1.001	0.943	1.062	0.983
Confrontation naming	1.018	0.960	1.080	0.551	1.025	0.985	1.066	0.228
Hypophonia								
Immediate recall	0.913	0.755	1.105	0.351	0.934	0.809	1.079	0.353
Delayed recall	1.214	1.029	1.432	0.022	1.070	0.897	1.276	0.451
Attention	0.851	0.664	1.092	0.206	0.83	0.715	0.964	0.016
Processing speed	1.009	0.996	1.022	0.183	1.001	0.993	1.009	0.770
Executive function	1.004	0.995	1.012	0.426	1.001	0.996	1.007	0.620
Verbal fluency	1.006	0.888	1.141	0.923	0.979	0.899	1.066	0.617
Confrontation naming	0.980	0.901	1.066	0.642	0.983	0.938	1.029	0.460

**Bold** denotes statistical significance.

**Table 5:** Interaction of covariates with the effect of cognitive performance on the global motor variable (the presence of at least one motor sign). Effect sizes (OR) with precision estimates [95% confidence interval lower (LL) and upper limits (UL)] are presented for each interaction term.

Cognitive Domain	Original Dataset				Imputed Datasets			
	OR	LL	UL	*p* Value	OR	LL	UL	*p* Value
Sex—male sex was used as reference								
Female sex by processing speed	1.001	0.995	1.006	0.813	0.999	0.995	1.003	0.650
Female sex by executive function	0.999	0.997	1.002	0.647	1.000	0.998	1.002	0.945
Race—Caucasian race was used as reference								
African American race by processing speed	0.999	0.991	1.007	0.766	1.003	0.997	1.009	0.365
Asian race by processing speed	1.004	0.975	1.035	0.773	1.004	0.986	1.023	0.667
Other race by processing speed	0.997	0.981	1.014	0.733	0.993	0.981	1.006	0.277
African American race by executive function	1.000	0.995	1.005	0.881	1.000	0.996	1.004	0.849
Asian race by executive function	1.002	0.989	1.015	0.800	1.004	0.993	1.014	0.481
Other race by executive function	1.000	0.988	1.013	0.957	1.002	0.994	1.011	0.619
*APOE*—*APOE2* was used as reference								
*APOE3* by processing speed	0.993	0.983	1.003	0.165	0.996	0.989	1.004	0.315
*APOE4* by processing speed	0.994	0.984	1.004	0.218	0.997	0.990	1.005	0.483
*APOE3* by executive function	1.001	0.997	1.006	0.532	1.001	0.996	1.005	0.797
*APOE4* by executive function	1.003	0.998	1.007	0.227	1.002	0.997	1.006	0.448
Years of age								
Age by processing speed	1.000	0.999	1.000	0.219	1.000	1.000	1.000	0.190
Age by executive function	1.000	1.000	1.000	0.499	1.000	1.000	1.000	0.411
Years of formal education								
Education by processing speed	0.999	0.999	1.000	0.090	1.000	0.999	1.000	0.331
Education by executive function	1.000	1.000	1.001	0.072	1.000	1.000	1.001	0.350
GDS								
GDS by processing speed	1.000	0.999	1.001	0.705	1.000	0.999	1.000	0.287
GDS by executive function	1.000	1.000	1.001	0.155	1.000	1.000	1.001	0.113
NPS—without NPS was used as reference								
Mild NPS by processing speed	0.999	0.991	1.007	0.825	1.002	0.996	1.009	0.500
Moderate-severe NPS by processing speed	1.000	0.992	1.008	0.950	1.002	0.996	1.008	0.547
Mild NPS by executive function	1.001	0.998	1.005	0.480	1.000	0.997	1.004	0.917
Moderate-severe NPS by executive function	1.002	0.998	1.006	0.265	1.001	0.997	1.004	0.737
CDR—the very mild stage was used as reference								
Mild stage by processing speed	1.001	0.995	1.008	0.671	1.000	0.995	1.005	0.993
Moderate stage by processing speed	0.990	0.981	0.999	0.027	0.995	0.988	1.002	0.177
Severe stage by processing speed	0.624	0.000	N/A	0.999	0.983	0.959	1.007	0.154
Mild stage by executive function	1.000	0.997	1.003	0.897	1.000	0.997	1.003	0.907
Moderate stage by executive function	1.000	0.995	1.006	0.889	0.998	0.994	1.002	0.312
Severe stage by executive function	N/A	N/A	N/A	N/A	1.000	0.985	1.015	0.993
Anxiolytics—the group without intake was used as reference								
Anxiolytics by processing speed	1.000	0.990	1.010	0.980	1.002	0.995	1.009	0.514
Anxiolytics by executive function	1.000	0.996	1.004	0.944	1.000	0.996	1.004	0.944
Antidepressants—the group without intake was used as reference								
Antidepressants by processing speed	1.004	0.998	1.009	0.228	1.002	0.998	1.006	0.318
Antidepressants by executive function	1.000	0.997	1.002	0.757	1.000	0.998	1.002	0.951
Antipsychotics—the group without intake was used as reference								
Antipsychotics by processing speed	0.999	0.986	1.012	0.886	1.001	0.993	1.008	0.876
Antipsychotics by executive function	1.003	0.996	1.011	0.408	1.001	0.994	1.008	0.837
FDA approved drugs for AD—the group without intake was used as reference								
Drugs for AD by processing speed	0.996	0.990	1.002	0.208	1.000	0.996	1.005	0.889
Drugs for AD by executive function	1.002	0.999	1.005	0.131	1.001	0.998	1.003	0.610

*APOE*: *apolipoprotein*; GDS: geriatric depression scale; NPS: neuropsychiatric score; CDR: clinical dementia rating scale; AD: Alzheimer’s dementia.

## Data Availability

For further information on access to the NACC database, please contact NACC (contact details can be found at https://naccdata.org/).

## Supplementary Materials

The following supporting information can be downloaded at: https://www.mdpi.com/article/10.3390/medicina61122116/s1, Table S1. Sensitivity analysis excluding severe dementia cases (CDR > 1). Table S2. Sensitivity analysis using the UPDRS-III total score as a continuous variable, based on the original (non-imputed) dataset.

## Abbreviations

The following abbreviations are used in this manuscript:

AD	Alzheimer’s dementia
NACC	National Alzheimer’s Coordinating Centre
UDS	Uniform Data Set
NIA/NIH	National Institute on Aging/National Institutes of Health
ADCs	Alzheimer’s Disease Centres
UPDRS-III	Unified Parkinson’s Disease Rating Scale Part-III
WMS-R	Wechsler Memory Scale—Revised
BNT-30	30-item version of the Boston Naming Test
DST	Digit Span Test
TMT-A	Trail Making Test—Part A
TMT-B	Trail Making Test—Part B
GDS	Geriatric Depression Scale
*APOE*	*Apolipoprotein E*
CDR	Clinical Dementia Rating
NPS	Neuropsychiatric severity score
SSRIs	Selective serotonin reuptake inhibitor
SNRIs	Serotonin and norepinephrine reuptake inhibitors
MAOIs	Monoamine oxidase inhibitors
NPI-Q	Neuropsychiatric Inventory Questionnaire
