# Supplementary material for: Associations Between Cognitive Performance and Motor Signs in Older Adults with Alzheimer’s Dementia
Source: Medicina (Kaunas). 2025 Nov 27;61(12):2116. doi: 10.3390/medicina61122116 (PMC12734484; doi:10.3390/medicina61122116)
Supplement: Supplementary file 1 [file medicina-61-02116-s001.zip › medicina-3939898-supplementary.pdf]

Supplementary Table S1 Sensitivity analysis excluding severe dementia cases (CDR>1).

Original dataset				
Cognitive domain	p-value	Odds Ratio	95% Confidence Interval Lower Limit	95% Confidence Interval Upper Limit
Episodic memory – immediate recall	0,931	1,002	0,962	1,044
Episodic memory – delayed recall	0,047	1,044	1,000	1,090
Attention	0,702	0,989	0,935	1,046
Processing speed	0,000	1,009	1,005	1,012
Executive function	0,023	1,002	1,000	1,003
Semantic verbal fluency	0,813	0,997	0,972	1,023
Confrontation naming	0,193	1,013	0,994	1,032
Imputed dataset				
Cognitive domain	p-value	Odds Ratio	95% Confidence Interval Lower Limit	95% Confidence Interval Upper Limit
Episodic memory – immediate recall	0,821	1,004	0,970	1,039
Episodic memory – delayed recall	0,013	1,048	1,010	1,088
Attention	0,060	0,957	0,915	1,002
Processing speed	0,000	1,007	1,004	1,009
Executive function	0,036	1,002	1,000	1,003
Semantic verbal fluency	0,574	0,994	0,974	1,015
Confrontation naming	0,088	1,013	0,998	1,028

Supplementary Table S2 Sensitivity analysis using the UPDRS-III total score as a continuous variable, based on the original (non-imputed) dataset.

Cognitive domain	p-value	β-coefficient	95% Confidence Interval Lower Limit	95% Confidence Interval Upper Limit
Episodic memory – immediate recall	0,376	0,035	-0,043	0,113
Episodic memory – delayed recall	0,014	0,105	0,021	0,188
Attention	0,866	0,009	-0,096	0,115
Processing speed	0,000	0,020	0,014	0,027
Executive function	0,018	0,003	0,001	0,006
Semantic verbal fluency	0,215	-0,031	-0,079	0,018
Confrontation naming	0,102	0,030	-0,006	0,067
